# Evaluation of a Tailored Multifaceted Pharmaceutical Care Intervention to Optimize Chronic Obstructive Pulmonary Disease Management: Protocol for a Cluster Randomized Controlled Trial

**DOI:** 10.2196/82806

**Published:** 2026-01-15

**Authors:** Xuedi Ma, Xiaocong Li, Xinyi Li, Hui Chen, Yang Wang, Xuan Jia, Yanyan Zhao, Wei Li, Lihong Liu

**Affiliations:** 1 Fuwai Hospital, National Center for Cardiovascular Diseases, Peking Union Medical College & Chinese Academy of Medical Sciences Beijing China; 2 Department of Pharmacy, China-Japan Friendship Hospital Beijing China

**Keywords:** cluster randomized controlled trial, chronic obstructive pulmonary disease, pharmaceutical care, health-related quality of life, medication adherence

## Abstract

**Background:**

Despite the widespread use of inhalation therapy, patients with chronic obstructive pulmonary disease (COPD) frequently experience suboptimal disease control due to medication nonadherence, improper inhaler use technique, and inappropriate device selection, which collectively impair health-related quality of life (HRQoL). Pharmacist-led interventions may help address these gaps. Interventions based on the information-motivation-behavioral skills model and supported by digital tools can improve adherence and self-management. This study evaluates the efficacy of a multifaceted pharmaceutical care intervention for COPD delivered through digital tool support.

**Objective:**

The primary objective is to compare the change in HRQoL, measured using the St George’s Respiratory Questionnaire, between the intervention and control groups from baseline to 12 months. Secondary objectives are to assess changes in medication adherence (Test of Adherence to Inhalers), quality of life (EQ-5D-5L), COPD-related medical costs, and patient-reported pharmacy service experience.

**Methods:**

This 1-year cluster randomized controlled trial evaluates a multifaceted pharmaceutical care intervention in adults with moderate to very severe COPD (Global Initiative for Chronic Obstructive Lung Disease, stages 2-4) who have confirmed suboptimal inhaler practice but remain matched to an appropriate inhaler device based on peak inspiratory flow rate measured using a digital tool. In total, 34 hospital-based cough and wheeze pharmaceutical care clinics were recruited and randomized (1:1) to either an intervention or a control group. The target sample size is 15 patients per site. Participants in the intervention group will receive tailored support using electronic adherence monitoring, inhaler use technique assessments, and peak inspiratory flow rate to optimize device selection and self-management. Participants in the control group will receive usual pharmaceutical care. Descriptive statistics will be used to summarize participant characteristics and outcomes. Linear mixed effects models will be used to compare primary and secondary outcomes. Subgroup analyses will explore effects by age, sex, education level, place of residence, and smoking status. Pharmacy service survey data will be analyzed qualitatively.

**Results:**

The trial was registered on July 15, 2024. Recruitment started on November 9, 2024, and enrollment was completed by December 31, 2025. As of December 31, 2025, we enrolled 454 participants, of whom 16 (3.5%) had completed the 12-month follow-up. The trial is expected to be completed by December 31, 2026, with results planned for publication in 2027.

**Conclusions:**

This multifaceted, pharmacist-led pharmaceutical care intervention may provide a scalable model for improving COPD management and HRQoL. If effective, the digitally supported program, grounded in the information-motivation-behavioral skills model, could be implemented in more than 1000 cough and wheeze pharmaceutical care clinics nationwide.

**Trial Registration:**

Chinese Clinical Trial Registry ChiCTR2400086943; https://tinyurl.com/75a9phbw

**International Registered Report Identifier (IRRID):**

DERR1-10.2196/82806

## Introduction

### Background and Rationale

Chronic obstructive pulmonary disease (COPD) poses a substantial global public health burden because of its high prevalence and economic impact [1]. In China, approximately 100 million individuals are affected [2], and the country faces the largest projected COPD-related economic burden globally from 2020 to 2050 [3]. Without effective interventions, new COPD cases and deaths in China may increase 1.5-fold over the next 25 years [4].

Inhalation therapy, the cornerstone of COPD management, relies critically on correct device technique and sustained medication adherence to achieve optimal disease control [5,6]. However, suboptimal practices persist: inhaler use technique errors occur in 50% to 100% of patients [7-10], nonadherence rates range from 50% to 80% [7-10], and inappropriate device selection affects more than 80% of patients [11]. These factors collectively undermine treatment efficacy, leading to poor disease control and impaired health-related quality of life (HRQoL) [12-14]. Therefore, it is essential to ensure the appropriate choice of inhalation devices, provide training on inhaler use, and promote medication adherence in COPD management.

Pharmacist-led interventions offer promise in addressing these gaps. Meta-analyses confirm their effectiveness in improving inhalation techniques (risk ratio=1.85, 95% CI 1.57-2.17; *P*<.0001) and medication adherence (risk ratio=1.41, 95% CI 1.24-1.61; *P*<.0001) [15-19]. In addition, this approach has been found to be cost-effective, with a mean cost saving of £671.59 (US $906.65) per patient (95% CI −£1584.73 [−US $2139.39] to −£68.14 [−US $91.99]; *P*<.0001; GBP £1 [US $1.35]) [20]. Notably, interventions grounded in the information-motivation-behavioral skills (IMB) model that facilitates health behaviors show enhanced adherence outcomes [21,22]. Consequently, IMB model−based interventions may be worthy of clinical promotion and application [15]. Concurrently, digital tools (eg, real-time adherence monitoring and telehealth) have demonstrated efficacy in promoting self-management and HRQoL among patients with COPD [23-26].

Although previous studies have explored the clinical effectiveness and cost-effectiveness of medication management for COPD, several aspects need to be addressed in the future studies. First, there is limited research that explored the impact of medication management on HRQoL among patients with COPD in the hospital-based pharmaceutical clinic setting; existing studies have focused primarily on behavioral outcomes [15-20]. If such pharmacist-led interventions are to be implemented in clinical settings, it is crucial to comprehensively understand their impact on patients’ health. Second, insufficient attention has been paid to device-patient mismatch despite its high prevalence [27,28]. Third, integrated interventions that combine the IMB theory with digital tools for real-world implementation remain scarce. To improve disease management and deliver patient-centered care that enhances patients’ well-being, it is essential to leverage these advancements in medication management and to tailor approaches to address the most prevalent medication use issues.

### Objective

This cluster randomized controlled trial evaluates a digitally enhanced, multifaceted pharmaceutical care intervention delivered across 34 cough and wheeze pharmaceutical care (CWPC) clinics. This will be a parallel-group superiority trial with 1:1 allocation to the intervention and control arms. This study aims to assess the impact of intervention on HRQoL in patients with moderate to very severe COPD who have confirmed suboptimal inhaler practice but remain matched to an appropriate inhaler device. It addresses evidence gaps through theory-driven, digitally enhanced management of medication adherence, inhaler use technique, and device selection. The findings aim to support the broader implementation of multifaceted pharmaceutical care models for COPD across more than 1000 CWPC clinics in 29 provinces and municipalities in China.

## Methods

### Study Setting

The multicenter trial will be conducted in 34 certified CWPC clinics embedded in secondary or tertiary hospitals across mainland China. Participating sites are required to (1) hold CWPC accreditation with at least 2 licensed pharmacists trained in COPD management, (2) have access to all guideline-recommended inhalation therapies, and (3) have the capacity to implement protocolized interventions. A complete list of participating hospitals and their provinces and municipalities is provided in Table S1 in Multimedia Appendix 1. Thus, these accredited CWPC clinics, staffed by well-trained pharmacists, are well positioned to facilitate effective participant recruitment.

### Eligibility Criteria

Recruited participants must meet the eligibility criteria listed in Textbox 1.

**Inclusion and exclusion criteria for participants.**

**Inclusion criteria (all criteria must be met simultaneously)**

- Aged 18 years or older
- Diagnosed with chronic obstructive pulmonary disease, with severity of airflow obstruction from level 2 to level 4 (according to the Global Initiative for Chronic Obstructive Lung Disease grades) [6], defined as a forced expiratory volume in 1 second <80% of the predicted value after the use of bronchodilators (pulmonary function test results within 3 months could be used for screening)
- Suboptimal inhaler practice confirmed by at least 1 incorrect technique identified using a standard inhaler checklist
- Appropriate device-patient match (peak inspiratory flow rate–guided selection using digital sensors)
- Signed the informed consent form

**Exclusion criteria**

- Inability to obtain informed consent from the patient or family members
- Comorbidities precluding participation, including terminal illness (life expectancy <6 months), active tuberculosis or lung cancer, or cognitive or physical impairment affecting inhaler use
- Pregnancy or lactation
- Concurrent enrollment in other clinical trials
- Inability to complete the 12-month follow-up (eg, relocation plans)
- Other conditions in which the pharmacist who evaluated the participant is unable to comply with the trial

### Interventions

#### Intervention Arm

The intervention was systematically developed using the IMB model and comprises 7 core components mapped to this theoretical framework. The 7 components are introduction to therapeutic regimens, diversified information support, inhaler use technique training, medication adherence management, medication management using a diary booklet, follow‑up management, and interactive question-and-answer sessions (Table S2 in Multimedia Appendix 1). The digitally enhanced, multifaceted pharmaceutical care intervention is delivered during scheduled CWPC clinic visits at baseline and at months 1, 6, and 9 (Table 1). Each face-to-face intervention visit lasts approximately 30 minutes and follows a structured checklist (Table S2 in Multimedia Appendix 1).

**Table 1:** Schedule of enrollment, interventions, and assessments (SPIRIT: Standard Protocol Items: Recommendations for Interventional Trials).

Time point		Baseline (−60 to 0 d; T0)	Month 1 (−14 to +14 d; T1)	Month 3 (−14 to +14 d; T2)	Month 6 (−14 to +14 d; T3)	Month 7 (−14 to +14 d; T4)	Month 9 (−14 to +14 d; T5)	Month 10 (−14 to +14 d; T6)	Month 12 (−14 to +14 d; T7)
**Enrollment**									
	Eligibility screen	✓							
	Informed consent	✓							
	Allocation (cluster level)	✓							
**Interventions**									
	Intervention arm	✓	✓		✓		✓		
	Control arm: standard CWPC^a^ pharmaceutical care	✓	✓		✓		✓		
**Assessments**									
	Visit date	✓	✓	✓	✓	✓	✓	✓	✓
	Telephone follow-up for questionnaire administration			✓		✓		✓	
	Basic information	✓							
	Medical history	✓							✓
	Physical examination	✓							
	Pulmonary function test	✓							✓
AECOPD^b^ status		✓	✓		✓		✓		✓
SGRQ^c^		✓	✓		✓		✓		✓
TAI-10^d^		✓	✓		✓		✓		✓
EQ‑5D‑5L^e^		✓							✓
COPD^f^ medication use in the past 2 wk		✓	✓		✓		✓		✓
COPD medication regimen at current visit		✓	✓		✓		✓		✓
Other concomitant medications in the past 2 wk		✓	✓		✓		✓		✓
Treatment costs of COPD		✓	✓		✓		✓		✓
Disease status			✓		✓		✓		✓
Pharmacy service survey									✓

^a^CWPC: cough and wheeze pharmaceutical care.

^b^AECOPD: acute exacerbations of chronic obstructive pulmonary disease.

^c^SGRQ: St George’s Respiratory Questionnaire.

^d^TAI-10: Test of Adherence to Inhalers.

^e^EQ‑5D‑5L: European Quality of Life-5 Dimensions-5 Level version.

^f^COPD: chronic obstructive pulmonary disease.

**Figure 1:**
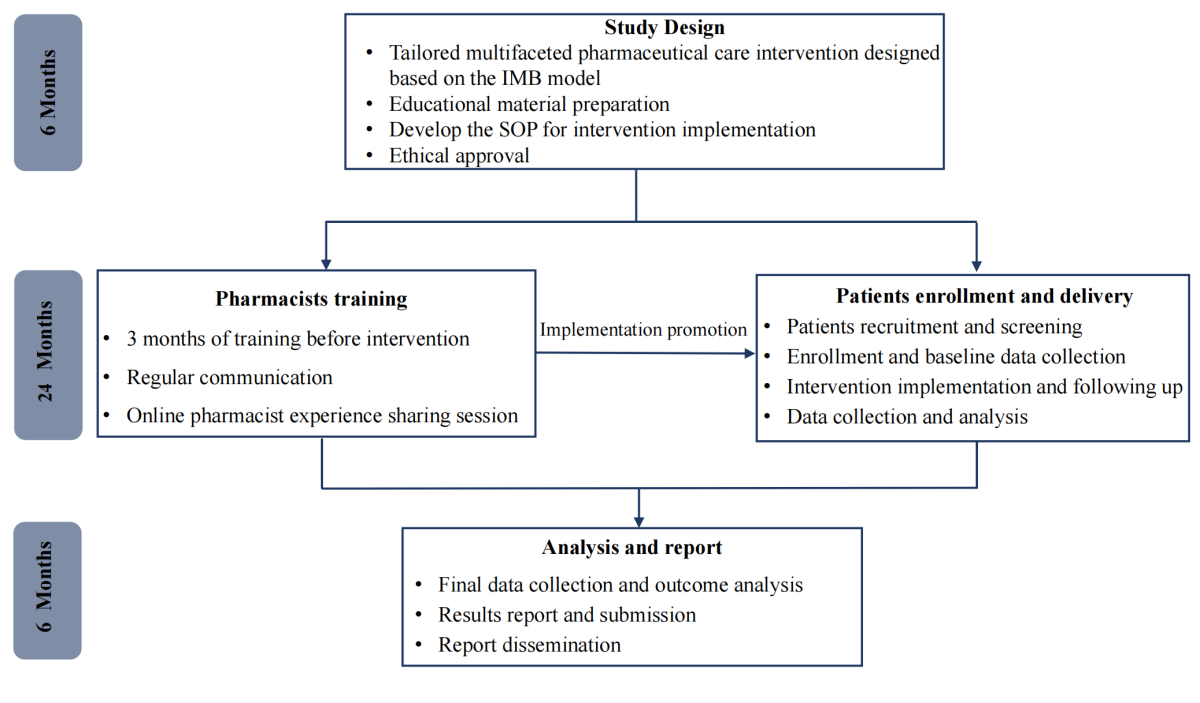
Overview of the study stages and implementation timeline. IMB: information-motivation-behavioral skills; SOP: standard operating procedure.

**Figure 2:**
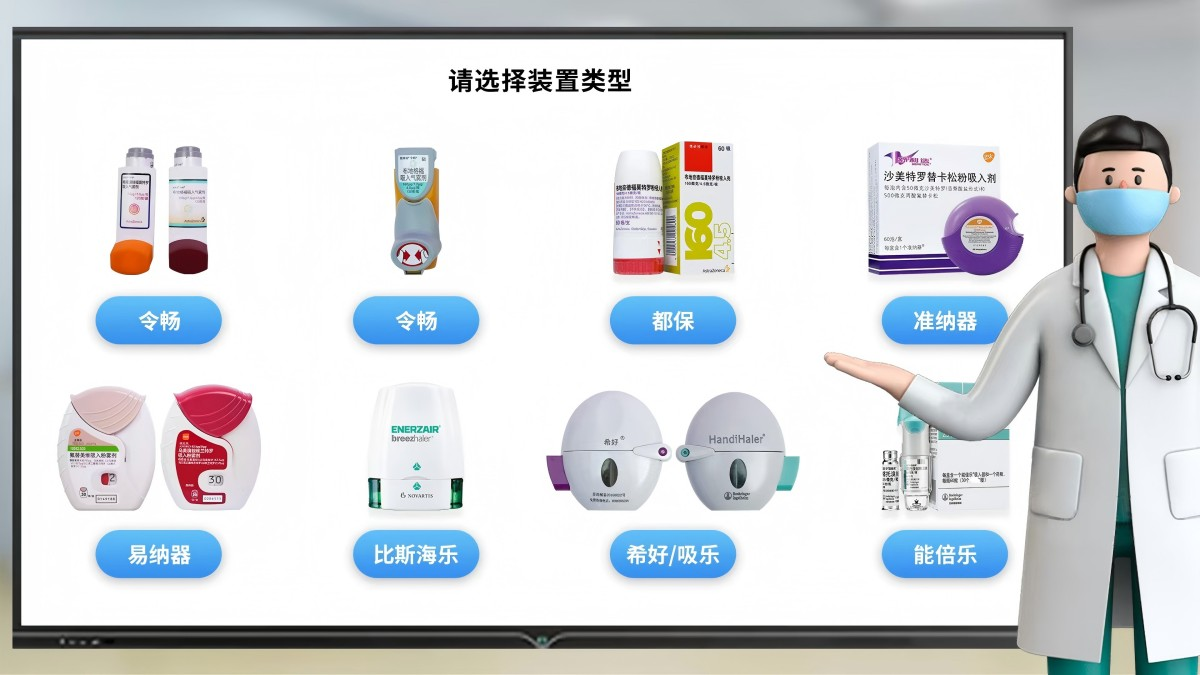
Illustration of the selection of digital inhaler sensor devices for inhaler use technique training.

Clinical pharmacists at intervention sites will receive standardized training before study initiation (Figure 1), including instruction on the trial rationale and objectives, COPD pharmacotherapy and inhaler use, and detailed guidance on implementing each intervention component. Pharmacists in the intervention arm will also be trained to use the digital inhaler sensor and the peak inspiratory flow rate assessment system to support inhaler use technique training (Figure 2) and device selection (see method 1 in Multimedia Appendix 1).

#### Control Arm: Standard CWPC Pharmaceutical Care

Sites allocated to the control arm will continue to provide standard CWPC pharmaceutical care according to existing practice. Pharmacists at participating sites will be trained on the standardized operating procedures (SOPs) corresponding to their allocation (Table S3 in Multimedia Appendix 1). At baseline and at months 1, 6, and 9, pharmacists will typically spend approximately 10 minutes per consultation (Table 1). Standard care includes (1) introduction to the therapeutic regimen (drug names, indications, and basic dosing instructions); (2) counseling on dosage and administration; (3) reminders regarding key drug precautions and management of common adverse drug reactions; and (4) demonstration and training in correct inhaler use technique using routine educational tools.

#### Strategies to Improve and Monitor Adherence to Intervention Protocols

To promote consistency of care within each study arm and to minimize contamination between arms, SOPs have been developed for both the intervention and control pharmaceutical care pathways (Tables S2 and S3 in Multimedia Appendix 1). Pharmacists at all participating sites will be trained in the SOPs corresponding to their allocation.

During the trial, completion of each digital-enabled pharmaceutical care component (intervention arm) or standard care element (control arm) will be documented in the electronic data capture (EDC) system using structured checklists (Tables S2 and S3 in Multimedia Appendix 1), which serve as fidelity-monitoring tools. The coordinating center will conduct periodic remote audits of EDC entries and, when necessary, on‑site or online supervision visits to provide feedback and reinforce protocol adherence. For patients, adherence to scheduled visits will be supported through automated appointment reminders delivered via the WeChat mini‑program.

#### Concomitant Care and Cointerventions

All COPD pharmacological treatments, including the choice and adjustment of inhalation therapies, will be prescribed at the discretion of the treating physicians in accordance with current Global Initiative for Chronic Obstructive Lung Disease (GOLD) guidelines and national guidelines. Other routine medical care for COPD and comorbid conditions (eg, vaccinations, pulmonary rehabilitation, and management of cardiovascular disease or diabetes) will be allowed and recorded as concomitant treatments.

### Study Outcomes

#### Primary Outcome

The St George’s Respiratory Questionnaire (SGRQ) will be used to assess HRQoL. The primary end point is the between-group difference in change from baseline to 12 months in the SGRQ total score, measured at baseline and 12 months after initiation of the intervention. The SGRQ was originally developed by Jones et al [29] and consists of 50 items divided into 3 sections: symptoms, activities, and impact. The scoring range is from 0 to 100, with a lower score indicating better health status in patients with COPD. SGRQ will be administered at baseline and at months 1, 6, 9, and 12 (Table 1).

#### Secondary Outcomes

##### Medication Adherence

The Test of Adherence to Inhalers (TAI-10) will be used to evaluate adherence to inhalation therapy. The main adherence outcome will be the between-group difference in change from baseline to 12 months in the TAI-10 score. The TAI-10 questionnaire was designed to assess the adherence to inhaler use in patients with asthma or COPD [30] and consists of 12 items. Items 1 to 10 assess the patient’s perspective and are scored on a 5-point scale (1=worst and 5=best). Items 11 and 12 reflect health care professional assessments and are scored on a 2-point scale (1=bad and 2=good). TAI assessments will be conducted according to the schedule shown in Table 1.

##### Generic Quality of Life

Generic health status will be assessed using the EQ-5D-5L, which covers 5 dimensions (mobility, self-care, usual activities, pain or discomfort, and anxiety or depression), each rated on 5 levels from *no problems* to *extreme problems* [31]. We calculated the EQ-5D-5L index using the value sets developed by Luo et al [32], with 1 representing the value of instrument-defined full health. The main EQ-5D-5L outcome will be the between-group difference in change from baseline to 12 months in the utility index. Measurement time points are shown in Table 1.

##### Pharmacy Service Survey

A pharmacy service survey will be used to assess patients’ perceived improvements in COPD-related and medication-related knowledge, self-reported quality of life, mastery of correct inhaler use after participation, and adequacy of communication with pharmacists (Table S4 in Multimedia Appendix 1). This survey will be administered at month 12 (Table 1).

##### Medical Cost

The medical cost will be collected using a microcosting approach [33] from the patient-level direct medical cost perspective over the 12-month trial period. At each scheduled visit, COPD-related direct medical expenses paid by patients will be recorded, including registration fees, examination costs, medication expenses, and other COPD-related medical service fees, when applicable. The primary economic outcome will be cumulative COPD-related direct medical costs per participant over 12 months, with the detailed data collection schedule provided in Table 1.

### Participant Timeline

The participant timeline is summarized in Table 1, which outlines the schedule of enrollment, assigned care delivery, and outcome assessments over the 12-month study period. A schematic overview of the study stages and timing is provided in Figure 1.

### Sample Size

The sample size was determined for the primary outcome, change in SGRQ total score from baseline to 12 months, using a cluster randomized superiority design for 2 means (PASS, version 2021; NCSS LLC). We assumed a between-group difference (intervention minus control) in SGRQ change of 4 units (the minimal clinically important difference), a common SD of 10 units, an intracluster correlation coefficient of 0.01, an average cluster size of 15 participants per community-based primary care clinic, 80% power, and a 2-sided α of .05. Under these assumptions, 8 clinics per arm (16 in total) were required. Allowing for 20% loss to follow-up, we planned to recruit at least 10 clinics per arm.

Ultimately, 34 clinics (17 per arm) were enrolled, with a target of 15 participants per clinic (planned N=510). Assuming up to 20% attrition (≥408 analyzable participants, approximately 204 per arm), this sample provides >90% power to detect a 4‑unit difference even if the intracluster correlation coefficient increases to 0.03. Details of the sample size formulas and calculations are provided in Multimedia Appendix 1 (see methods 2).

### Randomization and Blinding

Hospitals (clusters) will be stratified by their level (secondary or tertiary). Within each stratum, hospitals will be treated as clusters and randomized to either the intervention or the control group in a 1:1 ratio using simple random sampling. The randomization sequence will be generated by an independent statistician using a computerized random number generator. Randomization will be implemented centrally by the coordinating center. The allocation list will be kept in a password-protected file accessible only to the independent statistician and the designated trial coordinator. At each site, pharmacists will screen and enroll individual participants after cluster allocation has been assigned. Cluster allocation will be revealed to each participating hospital only after the data analysis has been completed.

Because of the nature of our interventions, the trial was registered as “single blind” (ChiCTR2400086943) presented in Multimedia Appendix 2. Pharmacists delivering the intervention cannot be blinded, and full participant blinding is not feasible because of differences in content and intensity of care. However, participants will not be explicitly informed of their clinic’s allocation. Outcome assessors and data analysts will remain blinded to group allocation. Group codes will be masked in the database, and analyses will be conducted using anonymized group labels (group A and group B) until the primary analysis is finalized. Unblinding of outcome assessors or data analysts is not planned during the trial.

### Data Collection and Management

Outcome, baseline, and other trial data will be collected at prespecified time points (Table 1). Data sources include hospital medical records and participant-reported questionnaires, administered in person at baseline and months 1, 6, 9, and 12, and by telephone at months 3, 7, and 10. Data will be recorded in an electronic case report form and stored in the EDC system by trained site pharmacists. EDC access will be role based and password protected.

Data quality will be promoted through standardized training on questionnaire administration and electronic case report form completion, the use of predefined variable definitions and built-in logic checks in the EDC system, and routine source data verification by an independent pharmacist at each site, supplemented by periodic central data monitoring and query resolution by the coordinating center. Standardized and validated instruments (SGRQ, TAI, and EQ-5D-5L) will be used as described earlier, and study-specific forms (pharmacy service survey) are provided in Table S4 in Multimedia Appendix 1.

To promote retention and complete follow-up, appointment reminders will be sent via the WeChat mini-program, and missed visits will be followed up by telephone. Participants who discontinue or deviate from the assigned care pathway will be encouraged to remain in the study for outcome assessment.

### Statistical Analysis

#### Baseline Characteristics

Baseline characteristics will be summarized by study arm using mean (SD) or median (IQR) for continuous variables and counts (%) for categorical variables. All statistical tests will be 2 sided, with a significance level of *P=*.05. Effect estimates will be reported with 95% CIs.

#### Analysis Populations

The primary analysis will follow the intention-to-treat principle, including all enrolled participants who provided informed consent, and analyzed according to the allocation of their recruiting clusters, regardless of intervention exposure or protocol deviations.

#### Primary Outcome Analysis

The primary end point (between-group difference in change from baseline to 12 months in SGRQ total score) will be analyzed using a linear mixed effects model accounting for clustering by site and adjusting for baseline SGRQ, cluster effects, and prespecified baseline characteristics. Treatment effect will be expressed as the adjusted between-group difference in change.

#### Secondary and Other Outcome Analysis

Continuous secondary outcomes (eg, TAI-10 and EQ-5D-5L utility index) will be analyzed using linear mixed effects models, adjusting for baseline value and cluster effects. Results of the pharmacy service survey will be summarized using descriptive statistics (proportions).

#### Subgroup Analyses

Subgroup analyses for the primary outcome will be conducted based on the following variables: age (<65 vs ≥65 years), sex (male vs female), education level (high school or below vs college or university or above), place of residence (rural vs urban), COPD severity (moderate vs severe vs very severe), smoking status (current or former smoker vs never smoker), and baseline number of acute exacerbations of COPD (0 vs ≥1). Interaction terms for group and these covariates will be included in the analysis.

#### Missing Data and Sensitivity Analyses

In the primary analysis, missing data for the primary outcome (SGRQ) will be handled using the last observation carried forward method. To assess the robustness of the primary outcome to different assumptions about missing data and analysis populations, several sensitivity analyses will be conducted, including (1) reanalysis of the primary outcome in the per‑protocol population, (2) complete‑case analysis without imputation, and (3) multiple imputation for missing SGRQ data.

#### Economic Evaluation

The EQ-5D-5L questionnaire will be used to obtain the health utility values for each participant before and after the intervention [32,34]. These values will be used to calculate the change in quality-adjusted life years (QALYs) over the 12-month follow-up for both groups [32,34]. The incremental cost-effectiveness ratio will be calculated as incremental cost divided by incremental QALYs. The willingness-to-pay per QALY threshold was set at 128,000 RMB (US $36,364) per QALY, equivalent to 1.76 times the gross domestic product per capita in China [35]. A 1-way sensitivity analysis will be conducted to assess the robustness of the model and the reliability of the results. All analyses will be performed using SAS (version 9.4; SAS Institute Inc).

### Data Monitoring and Quality Assurance

The trial will be overseen by a steering committee convened by the coordinating center (China-Japan Friendship Hospital), comprising clinical experts in COPD, pharmacologists, and epidemiologists. Given the low-risk nature of the intervention (pharmacist-led care enhancement) and the absence of planned interim efficacy analyses, a separate data monitoring committee will not be established. Trial monitoring will be conducted by the coordinating center’s monitoring team, which is independent of site investigators and reports to the steering committee.

The EDC system will automatically validate the input data. If the recorded values fall outside the acceptable range, a prompt will appear, requesting the user to double-check the entry. Each research center will have an independent pharmacist responsible for conducting source data verification. Additionally, the lead organization will provide real-time reminders to each center regarding participant enrollment and follow-up implementation plans. If any issues are identified during monitoring visits, the lead team will assist the site in resolving them. The lead organization will also conduct audits to assess the overall quality and completeness of the data, review source documents, interview investigators and coordinators, and ensure that clinical centers comply with the study protocol requirements.

Because the intervention is a pharmacist-led care enhancement, it is expected to pose minimal risk, and serious adverse events are unlikely. Nevertheless, potential risks will be reviewed throughout the trial. Study staff will be trained to identify and document any adverse events and to report them in accordance with each hospital’s standard procedures and ethical requirements.

### Ethical Considerations

This project has received approval from the Clinical Research Ethics Committee at the China-Japan Friendship Hospital, China, on May 20, 2024 (2024-KY-154). Researchers will provide participants or their legal representatives with an easily understandable informed consent form, approved by the ethics committee and allow them sufficient time to consider participation. Participants may not be enrolled in the study until a signed consent form is obtained from the participant or their legal representative. During the study, participants will be provided with all updated versions of the consent form and any relevant written information. The informed consent form will be retained as an essential document of the clinical trial for future reference.

All participant data were de-identified in the EDC system. Specifically, participants were identified using unique numerical codes rather than full personal identifiers. Each participant received compensation of CNY 200 (USD 28.66) for their time and travel expenses associated with study participation. Participants were informed during the informed consent process that they had the right to withdraw from the study at any time, for any reason, and without penalty.

### Dissemination Policy

The study findings will be disseminated to participating pharmacists, referring physicians, patients, and the broader medical community through peer-reviewed publications.

## Results

### Overview

The trial was registered on July 15, 2024 (ChiCTR2400086943). The study was funded in December 2024. Recruitment started on November 15, 2024, and enrollment was completed by December 31, 2025. As of December 31, 2025, we enrolled 454 participants, of whom 16 (3.5%) had completed the 12-month follow-up. The trial is expected to be completed by December 31, 2026, with results expected to be published in 2027 (Figure 1).

### Trial Status

The trial enrollment was completed by December 31, 2025, and the trial is now in the follow-up stage.

## Discussion

### Anticipated Findings

COPD remains a major public health challenge. The GOLD guideline highlights the importance of patient education, with particular emphasis on appropriate inhaler use technique and adherence to regular maintenance therapy [6]. Despite these recommendations, suboptimal disease control often persists due to 3 interrelated issues: medication nonadherence, inappropriate inhaler use technique, and inappropriate device selection [7-11]. These shortcomings not only exacerbate symptoms and hasten disease progression but also diminish HRQoL and increase health care facility use [12-14]. Against this backdrop, this study seeks to determine whether a multifaceted, pharmacist-led intervention, supported by digital tools and based on the IMB model, can mitigate these challenges in routine care. We anticipate that our digital-enabled pharmaceutical care program, which combines structured, IMB-guided pharmaceutical care with digital support via a WeChat mini-program and inhaler-integrated digital sensors, might yield clinically significant improvements in HRQoL.

### Comparison With Previous Work

Previous research has shown that pharmacist-led interventions can enhance critical aspects of inhalation therapy, including medication adherence and inhaler use technique [15-20]. IMB-based strategies have further demonstrated improved adherence in other chronic disease contexts by emphasizing clear information, meaningful motivation, and practical behavioral skills [21,22]. In this study, the *information* component involves interactive question-and-answer sessions, visual aids, and online educational resources designed to facilitate accessible and comprehensible knowledge. The *motivation* component enhances patient autonomy through regular follow-ups, feedback, and reinforcement of self-efficacy. The *behavioral skills* component underscores standardized training, digital sensor–based reminders, and real-time corrective guidance, all aimed at sustaining appropriate medication practices by promoting both correct medication administration and device appropriateness. Each of these 3 IMB dimensions is reinforced by quantifiable evaluation metrics, helping pharmacists deliver more precisely targeted management and education.

Meanwhile, digital tools such as real-time adherence monitoring and telehealth modules hold promise for improving self-management and HRQoL in patients with COPD [23-26]. In this intervention, *inhalable drug digital sensors* provide voice prompts to guide patients in the correct use of inhalers and record real-time inhalation behaviors. Pharmacists can intervene promptly when they identify incorrect use or recommend more suitable inhalation devices. In addition, the study expands beyond traditional face-to-face consultations by offering health knowledge updates, allowing participants to access project-specific information on COPD and medication use at home rather than in person. This approach leverages emerging technologies to establish a new model for pharmacist-led, multifaceted services and lays the groundwork for future digitally integrated pharmaceutical interventions.

### Strengths and Limitations

This trial has several anticipated strengths. It uses a multicenter, cluster randomized design across secondary and tertiary hospitals, which enhances external validity in higher-level care settings and reduces contamination between the intervention arm and control arm. The intervention is explicitly theory driven, grounded in the IMB model, and delivered by pharmacists who receive standardized training, which supports both intervention fidelity and future scalability. Furthermore, the study uses a comprehensive set of outcomes, including the SGRQ, TAI-10, EQ-5D-5L, and a pharmacy service survey, to capture not only clinical and behavioral effects but also patient-reported experiences with pharmacist-led care.

However, this study also possesses some limitations. First, our findings mainly reflect patients with COPD attending secondary and tertiary hospitals whose inhaler use technique is suboptimal and who are matched to an appropriate inhaler device, which may limit the generalizability of the results to other care settings. Future investigations should explore primary care settings to validate the intervention’s impact across multiple levels of the health care system. Second, we did not measure participants’ digital literacy, which could understate differences in engagement or create suboptimal outcomes among individuals who are less comfortable with technology. Finally, because this is a complex pharmacist-led behavioral intervention, neither the content nor the intensity of care can be fully masked to participants. Although the trial was registered as *single blind*, participants in the intervention arm are likely to be aware of receiving additional education and digital tools, and the primary and key secondary outcomes are all patient reported (SGRQ, TAI-10, EQ-5D-5L, and the pharmacy service survey). This may introduce performance and reporting bias. To mitigate this limitation, we used cluster randomization at the clinic level, applied an identical follow-up schedule and basic medication education and inhaler use training in both arms, and blinded the outcome assessors and statisticians.

### Conclusions

By facilitating the deep involvement of pharmacists in the medication management process for patients with COPD, the goal of the study is to improve medication adherence and correct inhaler use, thereby enhancing patient outcomes and reducing the disease burden associated with COPD. The study findings will be shared through peer-reviewed publications. If the study results demonstrate a positive impact of this digitally enhanced, multifaceted pharmaceutical intervention on COPD prognosis and disease burden, they will support the widespread adoption of this pharmaceutical management model in more than 1000 CWPC clinics across China, offering a novel approach to medication management for patients with COPD.

## Appendix

Multimedia Appendix 1 Trial stages, digital inhaler training, and trial registration dataset.

Multimedia Appendix 2 Trial registration: dataset.

## Abbreviations

COPD: chronic obstructive pulmonary disease
CWPC: cough and wheeze pharmaceutical care
EDC: electronic data capture
GOLD: Global Initiative for Chronic Obstructive Lung Disease
HRQoL: health-related quality of life
IMB: information-motivation-behavioral skills
QALY: quality-adjusted life year
SGRQ: St George’s Respiratory Questionnaire
SOP: standardized operating procedure
SPIRIT: Standard Protocol Items: Recommendations for Interventional Trials
TAI-10: Test of Adherence to Inhalers

## Data Availability

The datasets generated or analyzed during this study are not publicly available due to the inclusion of personal medication-related information but are available from the corresponding author on reasonable request.
