# Supplementary material for: Evaluation of a Tailored Multifaceted Pharmaceutical Care Intervention to Optimize Chronic Obstructive Pulmonary Disease Management: Protocol for a Cluster Randomized Controlled Trial
Source: JMIR Res Protoc. 2026 Jan 15;15:e82806. doi: 10.2196/82806 (PMC12856390; doi:10.2196/82806)
Supplement: Multimedia Appendix 1 [file resprot_v15i1e82806_app1.docx]

**Multimedia Appendix 1**

**Table S1. The hospitals included in the study**

| **Number** | **Hospitals** | **Provinces** |
| --- | --- | --- |
| 01 | Pizhou Traditional Chinese Medicine Hospital | Jiangsu Province |
| 02 | Tianjin Third Central Hospital | Tianjin City |
| 03 | Shaanxi Nuclear Industry 215 Hospital | Shaanxi Province |
| 04 | Nanyang Central Hospital | Henan Province |
| 05 | Jingmen Traditional Chinese Medicine Hospital | Hubei Province |
| 06 | Taiyuan Taihang Hospital | Shanxi Province |
| 07 | Gansu Provincial People's Hospital | Gansu Province |
| 08 | The First People's Hospital of Yunnan Province | Yunnan Province |
| 09 | Xiangya Changde Hospital | Hunan Province |
| 10 | Changzhou Second People's Hospital | Jiangsu Province |
| 11 | Chongqing Changshou District People's Hospital | Chongqing City |
| 12 | Nantong Traditional Chinese Medicine Hospital | Jiangsu Province |
| 13 | Shanghai Pudong New Area Gongli Hospital | Shanghai City |
| 14 | Yichang Yiling Hospital | Hubei Province |
| 15 | Wuxi Second People's Hospital (Affiliated Central Hospital of Jiangnan University) | Jiangsu Province |
| 16 | China-Japan Friendship Hospital | Beijing City |
| 17 | Shanghai Yangpu District Shidong Hospital | Shanghai City |
| 18 | The Fifth Affiliated Hospital of Zhengzhou University | Henan Province |
| 19 | Shangluo Central Hospital | Shaanxi Province |
| 20 | Weihai Municipal Hospital | Shandong Province |
| 21 | Huangshi Central Hospital | Hubei Province |
| 22 | Chongqing Liangjiang New Area People's Hospital | Chongqing City |
| 23 | The First People's Hospital of Taicang | Jiangsu Province |
| 24 | Sixth Affiliated Hospital of Xinjiang Medical University | Xinjiang Uygur Autonomous Region |
| 25 | Fifth People's Hospital of Chengdu | Sichuan Province |
| 26 | Shanxi Provincial People's Hospital | Shanxi Province |
| 27 | Jingzhou Central Hospital | Hubei Province |
| 28 | Xianyang Hospital of Yan'an University | Shaanxi Province |
| 29 | The First Hospital of Jilin University | Jilin Province |
| 30 | Suqian Hospital, Affiliated to Nanjing Drum Tower Hospital Group | Jiangsu Province |
| 31 | Ningde Municipal Hospital, Affiliated to Ningde Normal University | Fujian Province |
| 32 | The Second Affiliated Hospital of Guangzhou Medical University | Guangdong Province |
| 33 | Hebei General Hospital | Hebei Province |
| 34 | Nanfang Hospital, Southern Medical University | Guangdong Province |

**Table S2. The checklist of D‑PCARE multifaceted pharmaceutical care in the intervention group**

| **Procedure** | **IMB**  **module** | **Classification** | **Stage** | **Item** | **Whether complete** | **Intervention duration(min)** | **Note** |
| --- | --- | --- | --- | --- | --- | --- | --- |
| **1** | **Information** | **Introduction to therapeutic regimen** | -Baseline visit  -Center follow-up visit | **Medication Introduction.** Introduce the patients to the drug name |  |  |  |
|  |  |  | -Baseline visit  -Center follow-up visit | **Medication Introduction.** Introduce the patients to the pharmacological classification and indication |  |  |  |
|  |  |  | -Baseline visit  -Center follow-up visit | **Medication Introduction.** Introduce the patients to the clinical usage |  |  |  |
|  |  |  | -Baseline visit  -Center follow-up visit | **Medication Introduction.** Record the trade name and generic name of the drug in EDC system |  |  |  |
|  |  |  | -Baseline visit  -Center follow-up visit | **Instructions of usage and dosage.** Introduce the usage, dosage and frequency of the drug in detail |  |  |  |
|  |  |  | -Baseline visit  -Center follow-up visit | **Drug precautions.** Remind the patients of the precautions |  |  |  |
|  |  |  | -Baseline visit  -Center follow-up visit | **Education on taking medicine on time.** Patients are instructed to record whether they take medication on time in the diary booklet every day |  |  |  |
|  |  |  | -Center follow-up visit  -Telephone follow-up visit | **Medication administration record.** Record whether patients take medication on time/the number of times they do not take medication on time in the EDC system during the follow-up |  |  |  |
|  |  |  | -Baseline visit  -Center follow-up visit | **Adverse drug reaction management.** Explain the common adverse drug reactions |  |  |  |
|  |  |  | -Baseline visit  -Center follow-up visit | **Adverse drug reaction management.** Instruct the patients to record the symptoms of discomfort in the diary booklet in time |  |  |  |
| **2** | **Information** | **Diversified information support** | -Baseline visit | **Official accounts.** Confirm that the patient has followed the CWPC public account, and record this in the EDC system. |  |  |  |
|  |  |  | -Baseline visit | **Educational video.** Confirm that the patients understand how to search for the educational videos and other information in the public account/applet, and instruct the patients to study educational materials regularly, and record this in the EDC system |  |  |  |
|  |  |  | -Baseline visit  -Center follow-up visit | **Educational materials.** Hand over the educational brochure to the patients and introduce the content, and instruct the patients to learn it regularly |  |  |  |
|  |  |  | -Center follow-up visit  -Telephone follow-up visit | **Information Learning Record.** Record in the EDC system the number of times that the patients fail to study the materials during the follow-up period, and record the patients' reading status (such as the number of pages read) |  |  |  |
| **3** | **Behavioral skills** | **Inhaler usage technique training** | -Baseline visit  -Center follow-up visit | **Inhaler usage Demonstration.** Demonstrate the correct usage of the inhaler and record the inhaler type demonstrated in the EDC system |  |  |  |
|  |  |  | -Baseline visit  -Center follow-up visit | **Practical guidance.** Instruct the patients in practicing on the inhaler model. |  |  |  |
|  |  |  | -Baseline visit  -Center follow-up visit | **Patients practical exercise.** Ask the patient to demonstrate the use of inhalers, and observe the patients' operation and records the score of the patients' inhaler technique in the EDC system |  |  |  |
|  |  |  | -Baseline visit  -Center follow-up visit | **Evaluate whether the inhaler is operated correctly.** Evaluate whether the patients operate correctly with the help of digital sensors, and correct irregular drug use behaviors. The inhaler technique evaluation report will be provided to the patients for usage reference at home |  |  |  |
|  |  |  | -Telephone follow-up visit | **Record the Practice of Patients.** Record the patients' self-reported inhaler techniques in the EDC system |  |  |  |
|  |  |  | -Baseline visit  -Center follow-up visit | **Evidence for Medication Decision-Making.** Provide a report on the patients' inspiratory capacity (peak inspiratory flow rate) for physicians' reference in selecting the appropriate dosage |  |  | If the patient changed the dosage, please record. |
| **4** | **Motivation** | **Medication adherence management** | -Baseline visit  -Center follow-up visit | **Medication adherence assessment and management.** Evaluate the inhaler adherence and provide specific advice for patients by using the TAI questionnaire, and record the questionnaire score in the EDC system |  |  |  |
|  |  |  | -Baseline visit  -Center follow-up visit | **Medication adherence promotion.** Encourage patients to develop good medication habits (such as advising patients to combine medication habits with fixed activities of daily living) |  |  |  |
|  |  |  | -Baseline visit  -Center follow-up visit  -Telephone follow-up visit | **Medication reminder.** Inform that the WeChat mini program will push medication reminder regularly |  |  |  |
|  |  |  | -Baseline visit  -Center follow-up visit | **Medication inspection.** Ask the patients to bring the drugs used within the past 3 months or the empty bottles used up back for checking and recording the patients' drug use |  |  | Please record the patient's residual medicine at follow-up. |
| **5** | **Behavioral skills** | **Medication management with diary booklet** | -Baseline visit  -Center follow-up visit | **Providing medication diary booklet.** Provide the patients the medication diary booklet for documenting whether to take medication on time, whether discomfort occurs, and whether to learn the provided informational materials |  |  | Please record the date the diary booklet was issued. ______  ______  ______ |
| **6** | **Motivation** | **Follow-up management** | -Baseline visit  -Center follow-up visit  -Telephone follow-up visit | **Emphasize the importance of follow-up.** Emphasize the necessity of follow-up for monitoring medication efficacy and adjusting treatment as needed, and encourage the patients to follow the trial plan |  |  | Please record the patient's next visiting date. ______  ______ |
|  |  |  | -Baseline visit  -Center follow-up visit  -Telephone follow-up visit | **Regular follow-up reminder.** Remind the patients for regular follow-up through the WeChat mini program |  |  |  |
| **7** | **Information** | **Interactive Q&A sessions** | -Baseline visit  -Center follow-up visit | **Outpatient Q&A.** Encourage patients to ask questions and answer questions. |  |  | Please record questions. ______  ____________  ____________  ____________ |
|  |  |  | -Baseline visit  -Center follow-up visit  -Telephone follow-up visit | **Remote interaction.** Keep in touch with patients and answer patients' questions through WeChat mini-programs for additional communication |  |  |  |

**Signature.**

**Date.**

**Table S3 The checklist of standard CWPC pharmaceutical care in the control group**

| **Procedure** | **Classification** | **Stage** | **Item** | **Whether complete** | **Intervention duration(min)** | **Note** |
| --- | --- | --- | --- | --- | --- | --- |
| **1** | **Introduction to therapeutic regimen** | -Baseline visit  -Center follow-up visit | **Medication Introduction.** Introduce the patients to the drug name |  |  |  |
|  |  | -Baseline visit  -Center follow-up visit | **Medication Introduction.** Introduce the patients to the pharmacological classification and indication |  |  |  |
|  |  | -Baseline visit  -Center follow-up visit | **Medication Introduction.** Introduce the patients to the clinical usage |  |  |  |
|  |  | -Baseline visit  -Center follow-up visit | **Medication Introduction.** Record the trade name and generic name of the drug in EDC system |  |  |  |
|  |  | -Baseline visit  -Center follow-up visit | **Instructions of usage and dosage.** Introduce the usage, dosage and frequency of the drug |  |  |  |
|  |  | -Baseline visit  -Center follow-up visit | **Drug precautions.** Remind the patients of the precautions |  |  |  |
|  |  | -Baseline visit  -Center follow-up visit | **Adverse drug reaction management.** Explain the common adverse drug reactions |  |  |  |
| **2** | **Inhaler usage technique training** | -Baseline visit  -Center follow-up visit | **Inhaler usage Demonstration.** Demonstrate the correct usage of the inhaler |  |  |  |
| **3** | **Interactive Q&A sessions** | -Baseline visit  -Center follow-up visit | **Outpatient Q&A.** Answer questions of patients |  |  |  |

**Signature:**

**Date:**

**Table S4 Pharmacy Service Survey^*^**

| **Question** | **Yes** | **No** |
| --- | --- | --- |
| 1. **Did your participation in the study enhance your understanding of chronic obstructive pulmonary disease (COPD) and relevant medication knowledge?** |  |  |
| 1. **Did your self-perceived quality of life improve after participating in the study?** |  |  |
| 1. **Did you master the correct usage of inhalers under the guidance of pharmacists after participating in the study?** |  |  |
| 1. **Did you communicate fully with pharmacists during the study period?** |  |  |
| 1. **Which aspect of pharmaceutical care do you think was the most helpful to you?** |  | |
| 1. **Which aspect of pharmaceutical care do you think needs further improvement and enhancement?** |  | |

**^*^Questions 5 and 6 are open-ended questions, the pharmacists will document all responses provided by the patients.**

**Supplement method 1. Description of the digital tool, peak inspiratory flow rate (PIFR) assessment, and inhaler selection algorithm**
To enable standardized and objective assessment of inhalation technique and inspiratory capacity, we used an integrated digital toolset (**OHM-0**) consisting of two complementary components: (1) a **pressurized metered-dose inhaler (pMDI)**-mounted smart inhaler sensor to capture **hand-breath coordination** and inhalation **timing/duration** during aerosol inhaler use, and (2) a PIFR assessment system to quantify peak inspiratory flow and support inhaler suitability decisions. These measurements were used to guide individualized inhaler selection and pharmacist-led corrective training.


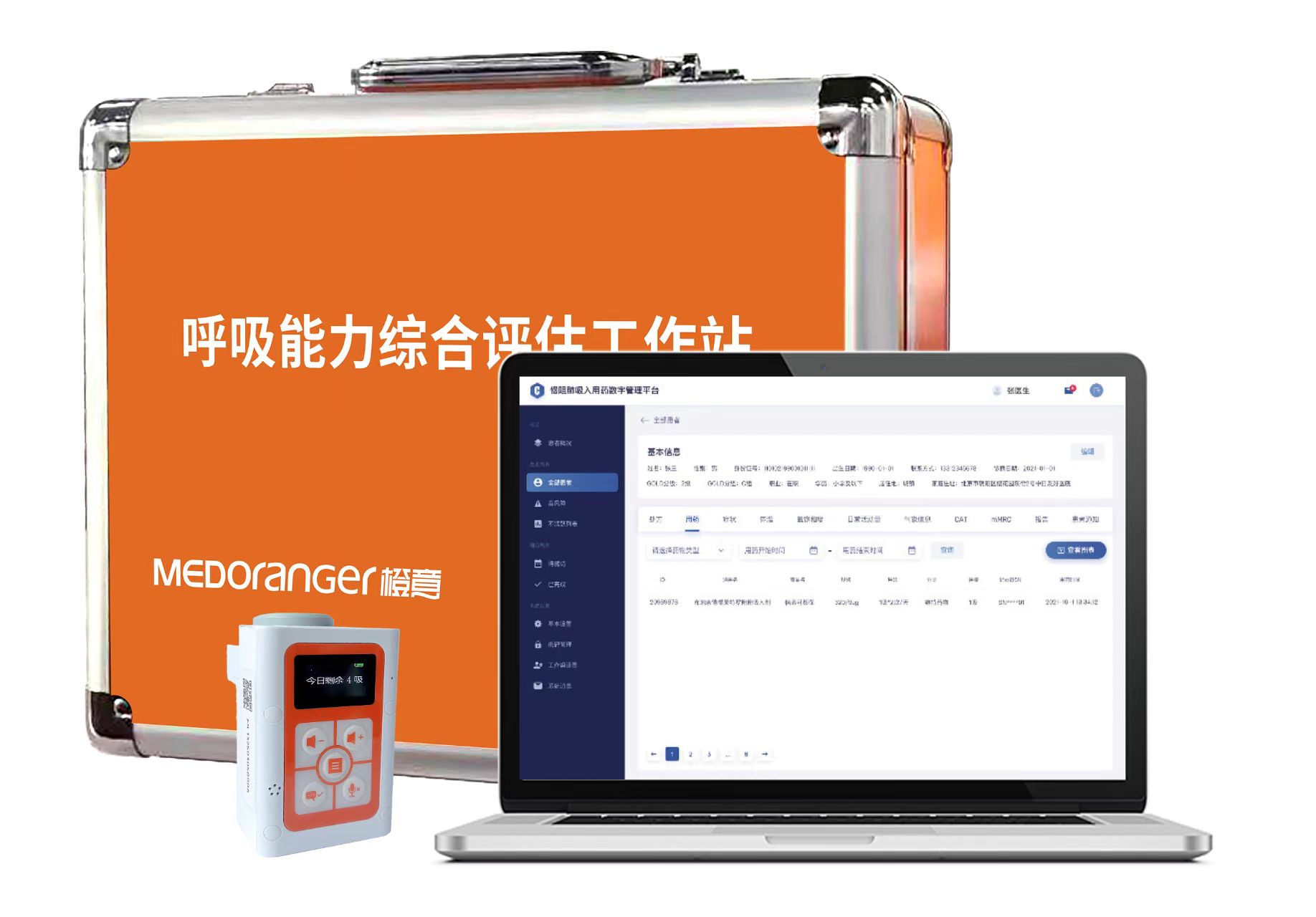


**Figure S1 Illustration of the integrated digital toolset (OHM-0)**

**1) pMDI-mounted smart inhaler sensor (Intelligent Inhalation Trainer)**
**Device name:** Intelligent Inhalation Trainer (pMDI-mounted smart sensor), manufactured by Chengyi Family Technology (Tianjin, China).
**Main functions:** The sensor is attached to pMDIs to objectively record the temporal relationship between inhalation and canister actuation, measure inhalation duration, and flag common technique errors (e.g., actuation without inhalation, delayed inhalation, insufficient inhalation time) via on-device alerts. It provides real-time audible feedback and transmits usage records to a backend system for subsequent review of inhaler technique.
**Validation/usage basis:** In this study, the sensor was used as an objective complement to checklist-based technique assessment, focusing on technique elements that are difficult to judge visually, particularly hand-breath coordination and the ability to sustain a slow, deep inhalation for **≥3 s**, thereby enabling targeted pharmacist feedback.

**2) PIFR assessment system (inhalation therapy comprehensive assessment workstation)**
**Device name:** Inhalation therapy comprehensive assessment workstation for PIFR testing, manufactured by Chengyi Family Technology (Tianjin, China).
**Main functions:** The system measures PIFR and can simulate internal resistance corresponding to different inhaler types, allowing assessment of whether inspiratory performance meets minimum requirements for effective drug delivery.
**How PIFR was performed:** Participants were seated comfortably and instructed to exhale fully, then initiate a rapid, forceful, and deep inhalation through the mouthpiece and maintain inhalation for approximately **2-3 s**. Three reproducible maneuvers were performed, and the bes**t PIFR value** was recorded for decision-making.
**Validation/usage basis:** PIFR results were used to support inhaler suitability assessment and training, and repeated assessments could be performed during follow-up to inform ongoing optimization.

**3) How measurements guided inhaler selection**
Inhaler selection was guided by an integrated algorithm combining PIFR, inhalation duration, and hand-breath coordination.

Patients with adequate coordination and **PIFR ≥30 L/min** were considered suitable for a broad range of inhaler types. When coordination was adequate but **PIFR <30 L/min**, inhalation duration was used to refine selection: patients able to sustain a slow inhalation for **≥3 s** were preferentially guided toward pMDIs and/or **soft mist inhalers (SMIs)**, whereas those with inhalation duration **<3 s** were recommended actively driven delivery systems. For patients with poor hand-breath coordination, device choice emphasized minimizing coordination demands: when **PIFR ≥30 L/min**, **dry powder inhalers (DPIs)** or actively driven devices were considered; when **PIFR <30 L/min**, those with inhalation duration **≥3 s** were guided toward SMIs or actively driven devices, while those unable to sustain inhalation were preferentially recommended actively driven delivery systems. Patients requiring assisted ventilation were directed to nebulized or other actively driven inhalation therapies.

**Supplement method 2. Sample size calculation**

**1. Design and hypothesis**

The primary outcome was the change in SGRQ total score from baseline to 12 months. The unit of randomization was the CWPC clinic (cluster-randomized design), with individual patients nested within clinics.

We planned a superiority trial comparing the mean change in SGRQ between the intervention and control arms. Let μ_I_ and μ_C_ denote the mean SGRQ change in the intervention and control groups, respectively. The null and alternative hypotheses were:

H₀: μ_I_ − μ_C_ = 0

H₁: μ_I_ − μ_C_ ≠ 0

The target difference (Δ) was 4 units, corresponding to the minimal clinically important difference (MCID) [35].

The calculation was performed using the “Test for Two Means in a Cluster-Randomized Design (Superiority by a Margin)” procedure in PASS 2021 (NCSS LLC), with the superiority margin set to 0 and a two-sided test.

Key assumptions:

- Expected between-group difference (Δ): 4 units
- Common SD (σ): 10 units (based on published SDs ≈ 8.6–12.1) [24, 36-40]
- ICC (ρ): 0.01
- Average cluster size (m): 15 participants per clinic
- Significance level (α): 0.05 (two-sided)
- Power (1 − β): 80%

**2. Individually randomized sample size**

For a continuous outcome in an individually randomized two-arm trial with equal allocation, the required sample size per group is:

$$n_{\text{indiv}}=\frac{2\sigma^{2}(Z_{1-\alpha/2}+Z_{1-\beta})^{2}}{\Delta^{2}}$$

where

- σ² is the common variance,
- Δ is the expected mean difference,
- $Z_{1-\alpha/2}$ is the standard normal quantile for a two-sided α,
- $Z_{1-\beta}$ is the standard normal quantile for the desired power.

Using α = 0.05 (two-sided, $Z_{1-\alpha/2}$ = 1.96), power = 80% ($Z_{1-\beta}$ = 0.84), σ = 10, and Δ = 4

Thus, if individuals were randomized (no clustering), approximately 98 participants per arm (196 total) would be required.

**3. Adjustment for clustering (design effect)**

For a cluster-randomized design with equal cluster size m and ICC ρ, the design effect (DE) is:

$$\text{DE}=1+(m-1)\rho$$

With m = 15 and ρ = 0.01:

$$\text{DE}=1+(15-1)\times0.01=1+0.14=1.14$$

The effective required sample size per group in the clustered design is:

$$n_{\text{clustered}}=n_{\text{indiv}}\times\text{DE}=98\times1.14\approx112\text{ participants per group}$$

The total number of participants required is therefore:

$$N_{\text{total}}\approx2\times112=224$$

Given an average of 15 participants per clinic, the required number of clusters is:

$$\text{Clusters total}\approx\frac{224}{15}\approx14.9$$

Thus, at least 15 clinics were required, which we rounded up to 16 clinics (8 per arm) to maintain balance. PASS 2021, using the “superiority by a margin” module for two means in a cluster-randomized design with these parameters, confirmed that 8 clinics per arm provide ≥80% power.

**4. Inflation for anticipated dropout**

To account for an anticipated 20% loss to follow-up at the participant level, we inflated the number of clinics:

- Planned minimum: ≥10 clinics per arm, each with 15 participants
- Nominal per-arm recruitment: 10 × 15 = 150 participants
- With 20% attrition: 150 × 0.80 = 120 analyzable participants per arm

This requires 20 clinics in total, with 300 recruited participants.

**5. Final sample size and post hoc assessment of power**

To enhance generalizability and statistical robustness, the final design included 34 clinics (17 per arm). With a target of 15 participants per clinic:

- Target total sample: 34 × 15 = 510 participants
- Assuming 20% attrition: 510 × 0.80 ≈ 408 analyzable participants (≈204 per arm)

We evaluated the robustness of power under a higher ICC (ρ = 0.03):

Design effect at ρ = 0.03 and m = 15

$$\text{DE}_{0.03}=1+(15-1)\times0.03=1+0.42=1.42$$

The effective per-arm sample size is:

$$n_{\text{eff}}=\frac{204}{1.42}\approx144\text{ participants per arm}$$

For comparison, the individually randomized sample size per arm required for 90% power (with the same α, σ, and Δ) is:

$$n_{\text{indiv,90\%}}=\frac{2\sigma^{2}(Z_{1-\alpha/2}+Z_{0.90})^{2}}{\Delta^{2}}$$

Our effective per-arm sample size (≈144) is greater than 131, indicating that the final design has >90% power to detect a 4‑unit difference in SGRQ change, even if ICC increases to 0.03.
