# Supplementary material for: Evaluation of a Tailored Multifaceted Pharmaceutical Care Intervention to Optimize Chronic Obstructive Pulmonary Disease Management: Protocol for a Cluster Randomized Controlled Trial
Source: JMIR Res Protoc. 2026 Jan 15;15:e82806. doi: 10.2196/82806 (PMC12856390; doi:10.2196/82806)
Supplement: Multimedia Appendix 2 [file resprot_v15i1e82806_app2.docx]

Trial registration: dataset.

| Category | Information |
| --- | --- |
| Primary registry and trial identifying number | ChiCTR2400086943 |
| Date of registration in primary registry | July 15, 2024 |
| Secondary identifying numbers | NA |
| Source(s) of monetary or material support | Self-sourced project |
| Primary sponsor | Clinical and Translational Medical Research Program of the Chinese Academy of Medical Sciences (grant no. 2024-I2M-C&T-B-092) |
| Secondary sponsor(s) | National High-Level Hospital Clinical Research Funding (grant no. 2023-GSP-GG-1), and the Special Project for the Development of Characteristic Disciplines of Fuwai Hospital, Chinese Academy of Medical Sciences (grant no. 2022-FWTS08) |
| Contact for public queries | hongllh@126.com |
| Contact for scientific queries | hongllh@126.com |
| Public title | Multifaceted Pharmaceutical Care Intervention to Optimize COPD Management |
| Scientific title | Evaluation of a Tailored Multifaceted Pharmaceutical Care Intervention to Optimize COPD Management: Protocol for a Cluster Randomized Controlled Trial |
| Countries of recruitment | China |
| Health condition(s) or problem(s) studied | Pharmaceutical care, Chronic obstructive pulmonary disease |
| Intervention(s) | Active comparator: This study was based on the IMB model to construct a multi-faced pharmaceutical intervention based on digital tools |
|  | Placebo comparator: Usual pharmaceutical care |
| Key inclusion and exclusion criteria | Ages eligible for study: ≥18 years  Sexes eligible for study: both  Accepts healthy volunteers: no |
|  | Inclusion criteria:   - Aged 18 years or older. - Diagnosed with COPD, and according to the GOLD grades29, the severity of airflow obstruction from level 2 to level 4, with a forced expiratory volume in 1 second (FEV1) less than 80% of the predicted value after the use of bronchodilators (pulmonary function testing results within three months could be used for screening). - Suboptimal inhaler practice confirmed by ≥1 of incorrect technique via standard inhaler checklist. - Device-person appropriate match (Peak Inspiratory Flow Rate (PIFR)-guided selection using digital sensors). - Signed the informed consent form. |
|  | Exclusion criteria:   - Unable to obtain informed consent from the patient or family members. - Pregnancy. - With terminal illnesses, malignant tumors, tuberculosis, cardiopulmonary failure, and those who cannot cooperate with pulmonary function testing due to their medical conditions. - Diagnosed with cognitive impairment, with physical limitations, or other conditions that affect inhalation usage technique. - With mobility limitations or other reasons that affect long-term follow-up. - Participating in other pharmaceutical intervention studies. - With other conditions in which the pharmacists evaluated the participant is unable to comply with the trial. |
| Study type | Interventional |
|  | Cluster randomization  Single blind |
|  | Primary purpose: To assess the impact of a comprehensive pharmaceutical care model on patients with moderate to very severe COPD, classified as GOLD stages 2 to 4. |
|  | Phase: NA |
| Date of first enrolment | November 9, 2024 |
| Target sample size | 300 |
| Recruitment status | Recruiting |
| Primary outcome(s) | The difference in health-related quality of life improvement between the two groups was assessed using the St. George’s Respiratory Questionnaire (SGRQ) at the 12-month endpoint. |
| Key secondary outcomes | Medication adherence (measured by the Test of Adherence to Inhalers, TAI-10), quality of life (assessed by the European Quality of Life-5 Dimensions-5 Level version, EQ-5D-5L), medical costs, and satisfaction with pharmaceutical care. |
